# Extremely fast construction and querying of compacted and colored de Bruijn graphs with GGCAT

**DOI:** 10.1101/gr.277615.122

**Published:** 2023-07

**Authors:** Andrea Cracco, Alexandru I. Tomescu

**Affiliations:** 1 Department of Computer Science, University of Verona, 37134 Verona, Italy;; 2 Department of Computer Science, University of Helsinki, Helsinki 00560, Finland

## Abstract

Compacted de Bruijn graphs are one of the most fundamental data structures in computational genomics. Colored compacted de Bruijn graphs are a variant built on a *collection* of sequences and associate to each *k*-mer the sequences in which it appears. We present GGCAT, a tool for constructing both types of graphs, based on a new approach merging the *k*-mer counting step with the unitig construction step, as well as on numerous practical optimizations. For compacted de Bruijn graph construction, GGCAT achieves speed-ups of 3× to 21× compared with the state-of-the-art tool Cuttlefish 2. When constructing the colored variant, GGCAT achieves speed-ups of 5× to 39× compared with the state-of-the-art tool BiFrost. Additionally, GGCAT is up to 480× faster than BiFrost for batch sequence queries on colored graphs.

de Bruijn graphs are one of the most fundamental data structures in computational genomics, appearing in countless applications, for example, assembly and analysis of sequencing data (Compeau et al. 2011) or RNA-seq data (Robertson et al. 2010), read error correction (Salmela and Rivals 2014; Limasset et al. 2020), alignment (Liu et al. 2016), compression (Benoit et al. 2015), and rearrangement detection (Cameron et al. 2017), just to name a few. To obtain a de Bruijn graph of order *k* for a multiset of strings (usually sequencing reads, or assembled genomes), for every *k*-mer in the strings, one adds an edge from the node corresponding to its prefix of length *k* − 1 to the node corresponding to its suffix of length *k* − 1. de Bruijn graphs usually also have associated an *abundance* threshold *a* so that edges (and thus nodes) are added only for *k*-mers appearing at least *a* times in the input strings.

de Bruijn graphs are appealing for several reasons. First, by increasing the abundance threshold, one can have a very simple but effective method of filtering out sequencing errors (i.e., erroneous *k*-mers). Second, having a graph structure allows for a smaller representation of the data in the presence of repeated regions, because equal substrings are represented only once in the graph. Third, by focusing on maximal *nonbranching* paths, that is, maximal paths whose internal nodes have in-degree and out-degree equal to one (also called *maximal unitigs*), one can discover “variation-free” regions.

Originally, maximal unitigs were introduced for the genome assembly problem; for example, assembly contigs are usually unitigs of a corrected assembly graph (Drezen et al. 2014; Ruan and Li 2020). However, by replacing each unitig with, for example, an edge labeled with the label of the unitig (label obtained by identifying the overlapping (*k* − 2)-mers), one gets an equivalent graph, but of much smaller size. Such a graph is usually called a *compacted de Bruijn graph*. Because the first and the last *k*-mer of every maximal unitig is a node of the compacted de Bruijn graph, it often suffices to compute only the strings labeling the maximal unitigs of a de Bruijn graph, not the actual graph structure. Moreover, reverse complements need special handling, which we describe in the Methods subsection “Preliminaries.” Many of the applications cited above actually use a compacted graph, owing to its equivalent representation power but significantly smaller size. In fact, if one just wants to represent the *k*-mers of a data set in plain text form, there exist more efficient equivalent representations (Břinda et al. 2021; Rahman and Medvedev 2021; Khan et al. 2022; and, even optimal, Schmidt and Alanko 2022; Schmidt et al. 2023), but all of these use maximal unitigs as a starting point.

A popular variant of a de Bruijn graph is the *colored* de Bruijn graph, originally introduced for de novo assembly and genotyping of variants (Iqbal et al. 2012). Such a graph is built from a *collection* of data sets, for example, different sequencing data sets or different (full) genome sequences. For every *k*-mer, colored de Bruijn graphs also store the identifiers (*colors*) of the data sets in which the *k*-mer appears. One can think of a colored de Bruijn graph as a compressed representation of the *k*-mers in a *collection* of data sets, but it retains enough information (i.e., the color of every *k*-mer) in order to identify each data set in this combined graph. Later applications include pangenomics (Zekic et al. 2018), RNA-seq quantification (Bray et al. 2016), bacterial genome querying (Luhmann et al. 2021), alignment and reference-free phylogenomics (Zekic et al. 2018), and microbiome research (Dufault-Thompson and Jiang 2022), just to name a few.

Although computing the maximal unitigs of a de Bruijn graph can be performed with a simple linear-time algorithm, the practical hardness of the problem stems from the fact that the initial de Bruijn graph does not fit the main memory in applications. Practical tools computing compacted de Bruijn graphs have to cleverly use the disk to store intermediary data, partition the data in order to use many CPU cores efficiently, and minimize CPU, RAM, and I/O bottlenecks. One of the first major tools for computing maximal unitigs was BCALM2 (Chikhi et al. 2016). BCALM2 first does a *k*-mer counting step inspired by KMC2 (Deorowicz et al. 2015) and a filtering pass based on the multiplicity of the *k*-mers. Then it finds *k*-mers that should be joined together by bucketing them on their left/right minimizers (corresponding to the minimizers of the leftmost and rightmost (*k* − 1)-mers) (Roberts et al. 2004). Each bucket is processed independently and in parallel to find all the possible extensions. Finally, BCALM2 glues all the unitigs that were produced in different buckets together using a union-find data structure.

The state-of-the-art tool for maximal unitig computation is Cuttlefish 2 (Khan et al. 2022). It starts with an initial *k*-mer counting step using KMC3 (Kokot et al. 2017). It uses a perfect hash computation on the *k*-mers using BBHash (Limasset et al. 2017). The key insight relies on a novel automaton-based approach to compute the branching state of every (*k* − 1)-mer, using only the minimum amount of information, that is, zero, one, or more than one left/right neighbors. Then, it builds the graph by looking at the automaton of every (*k* − 1)-mer, extending the unitig if the current (*k* − 1)-mer does not branch forward and the following (*k* − 1)-mer does not branch backward. Cuttlefish 2 tends to significantly write to disk to further resplit the intermediate buckets and keep the maximum memory usage low. Like BCALM2, it also does not compress the buckets on disk (except for prefix collapsing), and thus, very repetitive data sets still require large disk I/O. Moreover, for higher *k* values, KMC3 tends to use more time and memory, as it has to store the exact *k*-mers all the time, which, in consequence, also affects the behavior of Cuttlefish 2.

The state-of-the-art tool for maximal unitig computation with associated color information is BiFrost (Holley and Melsted 2020). It uses an in-memory only approach, with various blocked Bloom filters (Bloom 1970; Putze et al. 2010) partially indexed by minimizers that approximate the *k*-mers present in the final graph, and does several passes on the original input to remove the false edges wrongly created owing to the use of Bloom filters. Then it internally stores the *k*-mers grouped by minimizers, allowing for relatively fast deletions and insertions of new *k*-mers. However, although blocked Bloom filters are very memory efficient, they are not very cache efficient, even with the infra-block sse2 optimizations performed in BiFrost. Also, the memory representation of the *k*-mers gives a trade-off between the ease of doing small updates to the graph and the speed of inserting batches of *k*-mers; thus, the build time of the graph is still considerably high. To (optionally) build a colored graph, it uses various types of compressed bitmaps (roaring or simple bitsets) (Chambi et al. 2016) to store the set of colors of each *k*-mer. Although this allows fast insertions and querying, it stores redundant color sets information because *k*-mers that share the same set of colors are still encoded as two separate sets. We refer the reader to Section 7.5 of the survey (Chikhi et al. 2022) for other data structures for representing colored de Bruijn graphs.

In this paper, we present GGCAT, a software tool for efficient construction of compacted, and optionally colored, de Bruijn graphs, both in terms of running time and of memory usage; GGCAT also supports batched *k*-mer queries against a (colored) de Bruijn graph. In all these tasks, GGCAT is faster by at least an order of magnitude than the state-of-the-art tools.

## Results

### GGCAT overview

We propose a new tool for constructing compacted, and optionally colored, de Bruijn graphs, GGCAT. As opposed to BCALM2 and Cuttlefish 2, the first idea of GGCAT is to merge the *k*-mer counting step with unitig construction by adding a little more “context” information, which allows us to compute valid global unitigs inside each bucket that the input is split into. This avoids the storage of every single *k*-mer, because only unitigs built inside the buckets are written to disk. Moreover, as opposed to other tools, these unitigs are lz4-compressed before writing to disk, which allows for a substantial reduction in disk usage for highly repetitive data sets. Second, we avoid a union-find data structure (used by BCALM2) with a new joining step across buckets that guarantees exact results with very low *expected* running time. Third, we devise a parallelization pipeline that divides the algorithm into smaller execution units (e.g., reading from disk, *k*-mer counting, *k*-mer extension), thus preventing core stalling owing to waiting for data and thus lowering the dependence on the speed of the RAM.

On the theoretical side, we give a string-based definition of maximal unitig in the presence of reverse complements (*canonical maximal unitig*; Definition 1) that (1) allows us to avoid introducing a heavy formalism based on, for example, bidirected de Bruijn graphs and (2) closely mimics our algorithm, thus leading to a simple proof of correctness. Moreover, because our unitigs are in an edge-centric graph, in the Supplemental Methods (see also Supplemental Figs. S1, S2), we prove that they are equivalent to node-centric unitigs in a node-centric graph, as used by, for example, BCALM2 (which we also confirm experimentally) (Chikhi et al. 2016), a result that we did not find in the literature and may be of independent interest.

For colored graphs, we extend our algorithm above with an approach inspired by BiFrost but with several optimizations that allow comparable color map sizes with substantially improved build times. The main difference from BiFrost is that, instead of using an individual (compressed) color bitmap for each possible *k*-mer, GGCAT maps each color set to a *color set index*, an approach similar to, for example, Almodaresi et al. (2017), Pandey et al. (2018), and Mäklin et al. (2021). Moreover, to store each color set, we compute the difference between consecutive colors and compress them using a run-length encoding. Finally, when storing to disk, the color set indices of the consecutive *k*-mers of each unitig are also run-length encoded. This strategy proves efficient because unitigs are “variation-free” and thus usually have few color set indices associated to their *k*-mers. Because Cuttlefish 2 is significantly faster than BiFrost (on noncolored graphs), these ideas, combined with our improvements over Cuttlefish 2, lead to a major speed up over BiFrost for colored graphs.

Similar to BiFrost (Holley and Melsted 2020), GGCAT also supports querying the produced colored graph against batch input sequence queries. More precisely, for every query sequence in the uncolored case, we need to return the numbers (equivalently, percentage) of *k*-mers of the sequence that also appear in the entire target graph. In the colored case, for every color *c*, we need to return the number of *k*-mers of the query sequence matching *k*-mers of the graph that are colored with *c*. In practice, we need to query many input sequences at the same time (e.g., a FASTA file). GGCAT solves both types of batch queries by an approach very similar to the graph construction steps.

For *k* ≤ 64, GGCAT represents *k*-mers exactly. However, in some applications, larger values of *k* are needed. For example, for longer Illumina reads, the latest version of the SPAdes assembler (Prjibelski et al. 2020) recommends also *k* ∈ {77, 99, 127}. For long HiFi reads, the LJA assembler (Bankevich et al. 2022) uses values of *k* up to 5001. For comparing microbial genomes, the synteny block finder Sibelia (Minkin et al. 2013) uses *k* up to 5000. To support values larger than 64, GGCAT uses a nonbijective 128-bit Rabin–Karp hash function to represent each *k*-mer (where each of the four bases is represented by a different prime number) to avoid storing it in full length. In extremely rare cases, it can lead to some collisions in hash values that can cause unwanted joining of some unitigs or extra splittings of a maximal unitig. GGCAT can detect (but not correct) most of the collisions, warning the user if some errors can be expected in the graph. In all the tested data sets with *k* > 64, we found no occurrence of a hash collision.

GGCAT is written in Rust, and it is usable either as a stand-alone command line tool or with an API that supports both building and querying. The API is callable and supported in both Rust and C++ to allow easy integration with other tools. For example, the GGCAT C++ API for colored graph construction has already been successfully integrated by the Themisto pseudoalignment tool v3 (Alanko et al. 2023). When used as part of a pipeline to characterize pathogen competition and colonization dynamics in a longitudinal cohort of neonatal gut microbiomes (Mäklin et al. 2022), Themisto index construction using GGCAT is seven times faster than in v2.1 of Themisto (Mäklin et al. 2021; T. Mäklin, pers. comm.).

Finally, GGCAT also integrates the matchtig (Schmidt et al. 2023) and eulertig (Schmidt and Alanko 2022) Rust libraries, thus (optionally) computing also minimum plain-text representations of the set of *k*-mers of the input FASTA files.

### Tested tools, data sets, and hardware

To compute compacted de Bruijn graphs, we chose to compare only against Cuttlefish 2, because the article introducing it (Khan et al. 2022) showed that it significantly outperforms popular tools such as BCALM2 (Chikhi et al. 2016) and BiFrost (in its noncolored variant) (Holley and Melsted 2020) or other tools such as deGSM (Guo et al. 2021). To compute colored de Bruijn graphs, we chose to compare only against BiFrost, because the article introducing it (Holley and Melsted 2020) showed that it significantly outperforms popular tools such as VARI-merge (Muggli et al. 2019). We decided to not compare against Cuttlefish (Khan and Patro 2021) for colored graphs because it adopts a different convention for colors (each unitig can have only one subset of colors) and does not support querying the resulting graph. We run all tools in their default settings (for the commands used, see Supplemental Methods).

For the uncolored case, we use an Illumina whole-genome sequencing Human read data set, a Human gut microbiome read data set, 309,000 (309 K) *Salmonella* genome sequences, and 649,000 (649 K) Bacterial genomes. For the colored case, we use 100 Human genome sequences, 100,000 (100 K) *Salmonella* sequences from the full 309 K *Salmonella* data set (to save computational resources), and all Bacterial genomes. See the section “Software availability” for accession details and Table 1 for structural characteristics of these data sets. For the read data sets, we use an abundance threshold of two, and for the genome reference data sets, we use an abundance threshold of one. For a sanity check, we checked that for GGCAT the maximal canonical unitigs are exactly equivalent to the ones produced by BCALM2, for the uncolored graphs produced from 1000 *Salmonella* genomes, and from the Human read data set.

**Table 1.**
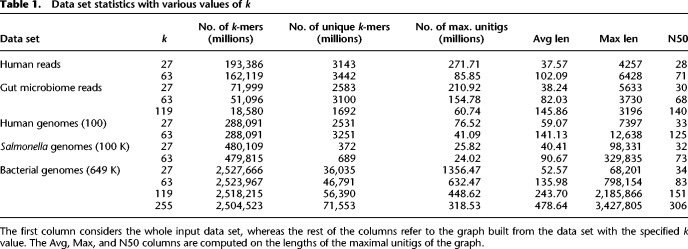
Data set statistics with various values of *k*

We ran the experiments on three servers of increasing power: a *small* server with an AMD Ryzen 5 3600 six-core CPU, 64 GB RAM, and a RAID 0 with two 7200 RPM HDDs; a *medium* server with an AMD Ryzen Threadripper PRO 3975WX 32-core CPU, 512 GB RAM, and a RAID 5 7200 RPM HDD; and a *large* server with two AMD EPYC 7H12 64-core CPUs, 2 TB RAM, and a SATA SSD.

### Construction results

In the uncolored case, for the Human read data set, we run two realistic values of *k*, 27 and 63. On the other three data sets used in the uncolored case, we tested the behavior for larger *k* values, where the graphs still remain complex: for gut microbiome reads, *k* = 119, and for the 309 K *Salmonella* genomes and 649 K Bacterial genomes, *k* ∈ {119, 255}. To save computational resources, we did not run Cuttlefish 2 for the latter data set for larger *k* values because the tool does not scale. We show the results in Table 2.

**Table 2.**
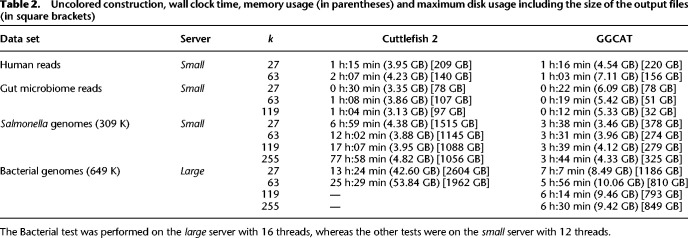
Uncolored construction, wall clock time, memory usage (in parentheses) and maximum disk usage including the size of the output files (in square brackets)

For Human reads and *k* = 27, GGCAT has a similar performance as Cuttlefish 2. However, for larger *k* values and on the other three larger data sets, GGCAT outperforms Cuttlefish 2 in terms of speed by up to 4.3× for *k* ≤ 63. The main speed improvements come from our new approach that merges the *k*-mer counting and unitig building steps into one unified step, which proves most useful for larger *k* values. For larger *k* values (119 and 255), GGCAT is faster than Cuttlefish 2 by up to 20.8×. (Notice also that, as opposed to GGCAT, Cuttlefish 2 does not support *k* values larger than 255.) Despite this, in all cases, GGCAT has an overall memory usage in the same order of magnitude as Cuttlefish 2 or one even substantially lower in the complex Bacterial genomes data set.

We also tested the scalability of GGCAT by computing the uncolored graph of an increasing number of *Salmonella* genomes (see Supplemental Fig. S3), the results showing a linear relation between the number of genomes and the running time.

The colored construction results are in Table 3. Compared with BiFrost, in the first data set GGCAT is 5.1× faster for *k* = 27 and 4.6× faster for *k* = 63. For the *Salmonella* genomes, for *k* = 27, GGCAT is 33.3× faster than BiFrost, and for *k* = 63, GGCAT is 39.3× faster than BiFrost. For *k* = 27, BiFrost crashed, whereas for *k* = 63, we stopped its run after 10 d to save computational resources. Instead, GGCAT completed both cases in under 14 h. The memory used by GGCAT in the colored construction tests is from 3.7× to 12× less than BiFrost, but this is not directly comparable because GGCAT uses disk intermediate storage and BiFrost uses a fully in-memory algorithm. Moreover, in Supplemental Table S1, we measure the size of the color index constructed by GGCAT and BiFrost (i.e., the set of colors of each *k*-mer). In the tested data sets, GGCAT's index is from 20 to two times smaller than that of BiFrost.

**Table 3.**
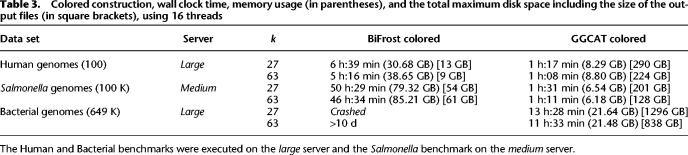
Colored construction, wall clock time, memory usage (in parentheses), and the total maximum disk space including the size of the output files (in square brackets), using 16 threads

Depending on the characteristics of the data set, the available disk space for temporary files should be from 2× to 7× the size of the input, whereas the main memory for uncolored construction should be at least 16 GB for medium-sized genome collections and 32 GB for large ones (GGCAT usually consumes significantly less memory than these amounts but can consume more if inputs with particular characteristics are passed to it). For uncolored construction, an empirical way to have a rough estimation of the size of the needed main memory is by dividing the input size by 30.

### Colored querying results

To test querying, we used the colored graphs of 100 Human genomes for *k* ∈ {27, 63} produced in the previous test, and queried them (cold runs) using 4 million 250-bp sequencing reads from the first data set of Human reads (first 4 million sequences from the D3_S1_L001_R1_008.fastq.gz file of the Human genome data set). Results (in Table 4) show that GGCAT outperformed BiFrost by 83.6× for *k* = 63, whereas for *k* = 27, GGCAT was more than 480× faster than BiFrost. This significant improvement is because we implement querying as a natural extension of the unitig construction step, thus benefiting from all its optimizations.

**Table 4.**
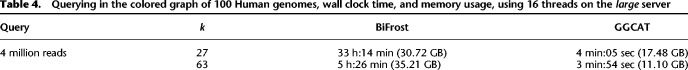
Querying in the colored graph of 100 Human genomes, wall clock time, and memory usage, using 16 threads on the *large* server

## Discussion

Computing a compacted de Bruijn graph (and optionally colored) is one of the most fundamental problems in computational genomics, with a long history of computational tools developed for this problem. GGCAT pushes the boundary not only in terms of a highly efficient implementation based on both novel algorithmic aspects (e.g., combining *k*-mer counting with unitig construction, and a new strategy of joining partial unitig across buckets) but also in terms of an efficient parallelization pipeline minimizing idle CPU cores and disk I/O bottlenecks, or further optimizations such as lz4-compression of data written to disk. GGCAT reduces sequence queries to an approach similar to graph construction, thus benefiting from its highly optimized architecture.

Overall, this leads to a several times improvement over Cuttlefish 2 (and even bigger for larger *k* values) and a two orders of magnitude improvement over BiFrost for colored construction and querying. GGCAT can thus have a significant impact in all downstream analyses that require computing a compacted de Bruijn graph.

One algorithmic limitation of GGCAT is that it is not optimized for very small values of *k* (i.e., *k* < 15). Moreover, like most disk-based de Bruijn construction tools, GGCAT's performance significantly depends on the disk and RAM speed. Thus, an NVME disk and a fast RAM are heavily preferred. GGCAT scales well in terms of number of threads, so for practical purposes, we recommend a CPU with at least eight cores/16 threads. Moreover, our experiments show that the suggestions above are valid for all the data sets we tested, that is, reads or references of small or large genomes, for both colored and uncolored construction. As future work, it would also be useful to extend GGCAT to support online updates, meaning updating the compacted colored de Bruijn graph with multiple small sets of reads or genomes while being able to query the graph between the updates.

## Methods

### Preliminaries

In this paper, all strings are over the same alphabet Σ = {*A*, *C*, *G*, *T*}. We denote concatenation of two strings *x* and *y* as *x* · *y*. If *x* is a substring of *y*, we write *x* ∈ *y*. We denote the length of a string *x* as |*x*|. Given a string *x* of length at least *k*, we denote by pre_*k*_(*x*) the prefix of *x* of length *k*; by suf_*k*_(*x*), the suffix of *x* of length *k*. For two strings *x* and *y* such that suf_*k*_(*x*) = pre_*k*_(*y*), we denote by $x{⊙}^{k}y$ the string *x* · suf_|*y*|−*k*_(*y*) (the *merge* of *x* and *y*). Given a set or multiset *R* of strings, we denote ends_*k*_(*R*) = {pre_*k*_(*x*), suf_*k*_(*x*):*x* ∈ *R*}.

A *k-mer* is a string of a given length *k* over the alphabet Σ. Given a *k*-mer *q*, we say that *q* is a *k*-mer of a string *x* if *q* ∈ *x* (in this case, we also say that *q appears*, or *occurs*, in *x*). Given a set or a multiset *R* of strings, we say that *q appears* in *R*, and write *q* ∈ *R* if *q* appears in some string in *R*. The *edge-centric* de Bruijn graph or order *k* of a multiset *R* of strings is defined as the directed graph having as nodes the (*k* − 1)-mers appearing in *R*, where we add an edge from a node *x* to a node *y* if suf_*k*−2_(*x*) = pre_*k*−2_(*y*) and $x{⊙}^{k-2}y\in R$. That is, the set of edges is exactly the set of all *k*-mers of *R*. In such an edge-centric graph, the *spelling* of a path *P* = (*x*_1_, …, *x*_*t*_) is the string $x_{1}{⊙}^{k-2}x_{2}{⊙}^{k-2}\cdots {⊙}^{k-2}x_{t}$.

A *unitig* is defined as a path of the de Bruijn graph of *R* containing at least one edge, such that all the internal nodes of the path (i.e., different from the first and the last) have an in-degree and an out-degree equal to one. In this paper, paths do not repeat nodes, except for possibly the first and the last, in which case we say that the path is a *cycle*. If two cycles *C*_1_ and *C*_2_ are such that *C*_2_ can be obtained from *C*_1_ by a cyclic shift, then we say that *C*_1_ and *C*_2_ are *equivalent*. A *maximal* unitig is one that cannot be extended by one node without losing the property that it is a unitig. We are interested in outputting the *set* of all maximal unitigs of a de Bruijn graph, where for unitigs that are cycles we need to output only one cyclic shift (i.e., in the output, there can be no equivalent cycles). Our definition of unitigs is for edge-centric graphs. For *node-centric de Bruijn graphs*, unitigs need to be defined by imposing an additional condition (see, e.g., Chikhi et al. 2016; Khan et al. 2022). To the best of our knowledge, we are not aware of a formal proof of equivalence between these types of unitigs in the two types of graphs. As such, we give this proof in the Supplemental Methods.

For ease of notation, by unitig we will also refer to its *spelling*. Clearly, the first and last node of a unitig different from a cycle must satisfy the condition that *either* its in-degree is different from one or its out-degree is different from one. Note that under this definition, the maximal unitigs form a partition of the edges, that is, of the *k*-mers of *R*.

For the rest of this paper, we consider an alternative definition of maximal unitigs that does not explicitly use a de Bruijn graph. This has several advantages: It connects to the recent literature on *spectrum preserving string sets* (unitigs being one such type of set) (Břinda et al. 2021; Rahman and Medvedev 2021; Schmidt et al. 2023); it naturally extends to reverse complements without introducing heavy definitions related to bidirected de Bruijn graphs; and, ultimately it matches our algorithm, which proceeds bottom-up by iteratively merging *k*-mers and unitigs as long as possible (i.e., the existence of branches in the de Bruijn graph is checked implicitly via *k*-mer queries).

Given a multiset *R* of strings, and a string *x*, we denote by occ(*x*, *R*) the number of occurrences of *x* in the strings of *R*, each different occurrence in a same string in *R* being counted individually. Given a string *x* ∈ Σ, we denote by *x*^−1^ ∈ Σ the reverse complement of *x*. Given a multiset *R* of strings and a string *x*, if *x* ≠ *x*^−1^, we define occ_cn_(*x*, *R*) = occ(*x*, *R*) + occ(*x*^−1^, *R*); otherwise, occ_cn_(*x*, *R*) = occ(*x*, *R*). We analogously define app(*x*, *R*) = min (1, occ(*x*, *R*)) and app_cn_(*x*, *R*) = min (1, occ_cn_(*x*, *R*)).

Given multisets of strings *R* and *U*, we say that *R* and *U* have the same *k-mer set* if any *k*-mer that appears in one of the sets also appears in the other set. Analogously, we say that *R* and *U* have the same *canonical k-mer set* if for any *k*-mer *q* that appears in one of the sets, it holds that *q* or *q*^−1^ appears in the other set. We can equivalently express the fact that sets *R* and *U* have the same noncanonical *k*-mer set with the condition

$$\forall q\in \Sigma^{k}\;\;\mathrm{occ}(q,R)\geq 1\;\mathrm{iff}\;\mathrm{occ}(q,U)\geq 1.$$

Likewise, *R* and *U* have the same *canonical k-mer set* if

$$\forall q\in \Sigma^{k}\;\;\mathrm{oc}c_{\mathrm{cn}}(q,R)\geq 1\;\mathrm{iff}\;\mathrm{oc}c_{\mathrm{cn}}(q,U)\geq 1.$$

We now give an equivalent *string-centric* definition of the *set U* of maximal unitigs of a multiset *R* of strings, under our formalism. As a warm-up, we start with the case when we do not have reverse complements.

First, we require that all strings in the *set U* have a length of at least *k*, meaning unitigs contain at least one edge. Second, we require that *R* and *U* have the same *k*-mer set. Third, if a (*k* − 1)-mer appears at least two times in *U*, then it cannot be an internal node in any unitig. In other words, we forbid merging two separate unitigs at a branching (*k* − 1)-mer, because such branching (*k* − 1)-mer must appear in at least one other string in *U*:

$$\begin{array}{rl} & \forall q\in \Sigma^{k-1}\;\mathrm{if}\;\mathrm{occ}(q,U)>1\;\mathrm{then}\;q\;\mathrm{appears}\;\mathrm{only}\;\mathrm{as}\;\mathrm{prefix}\;\mathrm{or}\;\mathrm{suffix}\\ & \mathrm{of}\;\mathrm{strings}\;\mathrm{in}\;U. \end{array}$$

Note that the above property also ensures that no two equivalent cyclic unitigs are in *U*.

To also impose maximality, we state that a (*k* − 1)-mer is a prefix or a suffix of a unitig if and only if it is either branching, a sink, or a source:

$$\forall q\in \mathrm{end}s_{k-1}(U),\;\;\underset{c\in \Sigma }{\sum }\;\mathrm{app}(q\cdot c,U)\neq 1\;\mathrm{or}\;\;\underset{c\in \Sigma }{\sum }\;\mathrm{app}(c\cdot q,U)\neq 1.$$

 Having defined maximal unitigs without reverse complements, we now give the string-centric definition of maximal unitigs also assuming reverse complements (which we call *canonical maximal unitigs*). In fact, we give a more general one, also handling a required abundance threshold of the *k*-mers in *R*, further underlining the flexibility of our string-centric view.

Definition 1 (canonical maximal unitigs). *Given a multiset R of strings and integers k* ≥ 2 *and a* ≥ 1*, we say that a* set *U of strings is the set of* canonical maximal unitigs *of R with k-mer size k and abundance threshold a if the following conditions hold:*

1. ∀x∈U*,* |*x*| ≥ *k, and* ∀x,y∈U*, x* ≠ *y*^−1^ *(note that x* ≠ *y is guaranteed by the fact that U is a set);*
2. $\forall q\in \Sigma^{k}\mathrm{oc}c_{\mathrm{cn}}(q,R)\geq a\;iff\;\;\mathrm{oc}c_{\mathrm{cn}}(q,U)\geq 1$ *(same canonical k-mer multiset, with abundances);*
3. $\forall q\in \Sigma^{k-1}$*, if*  occ_cn_(*q*, *U*) > 1, *then q and q*^−1^ *appear only as prefix or suffix of strings in U (unitigs do not span over branching* (*k* − 1)*-mers); and*
4. $\forall q\in \mathrm{end}s_{k-1}(U)$*,* $\sum_{c\in \Sigma }\mathrm{ap}p_{\mathrm{cn}}(q\cdot c,U)\neq 1\;or\;\sum_{c\in \Sigma }\mathrm{ap}p_{\mathrm{cn}}(c\cdot q,U)\neq 1$ *(maximality)*.

In our algorithm, we build unitigs incrementally, starting from individual *k*-mers (i.e., individual edges of the de Bruijn graph, which are unitigs) and extending them in both directions, as long as the resulting string remains a unitig (by checking for the satisfaction of Condition 3 in Definition 1 at each step, that is, whether we have reached the end of a unitig or not). Even though this is a simple strategy, behind other tools such as that of Chikhi et al. (2016), it is nontrivial how to implement this efficiently in terms of running time, memory consumption, disk usage, and parallelization.

Given an integer *m* ≤ *k* and a rolling function hash:Σ^*m*^ → ℤ, the *minimizer* of a *k*-mer *x* is

$$\mathrm{mini}(x)=\underset{y\in \Sigma^{m}\wedge y\in x}{\min}\;\mathrm{hash}(y).$$

Note that in this definition the minimizer is only a hash value and does not keep track of the particular position of the *m*-mer that has that minimum hash value. The *m* parameter is automatically chosen by GGCAT (it can also be user-defined) based on the value of *k*; for the precise function used to compute *m* from *k*, see Supplemental Table S2.

Throughout the algorithm, we will refer to *buckets* as a partition of the data that is stored as a single blob; for example, when stored on disk each bucket corresponds to a file. Multiple buckets are used to partition data in a way that is optimized for parallelization, thereby allowing for parallel and independent processing of each bucket. They are also used to reduce the memory consumption of the algorithm because only the buckets that are currently being processed occupy main memory, whereas the other ones use only disk space.

In the rest of this section, we present the algorithm for maximal unitigs without reverse complements, and then in the section “Construction correctness,” we explain the changes for canonical maximal unitigs.

### Read splitting

Each read *R*_*j*_ is split into substrings $S_{1},\ldots ,S_{\ell_{j}}$ that overlap on *k* − 2 characters, such that all (*k* − 1)-mers of *S*_*i*_ have the same minimizer, for all *i* ∈ {1, …, ℓ_*j*_}. For the minimizer hash function hash, we use the ntHash (Mohamadi et al. 2016) function because it can give fast computation while ensuring good randomness in its value. Note that we can have multiple minimizer locations in the same substring *S*_*i*_ as long as they have the same hash value. We can compute $S_{1},\ldots ,S_{\ell_{j}}$ in linear time in the size of *R*_*j*_ as follows. First, for every *m*-mer *x* of *R*_*j*_, we compute hash(*x*) in a rolling manner. Then, in a sliding window manner, we compute the minimum of each window of *k* − *m* consecutive *m*-mers (which correspond to a (*k* − 1)-mer). Finally, we group consecutive (*k* − 1)-mers that share the same minimum in their corresponding window. For efficiency, we perform these three steps in a single pass over *R*_*j*_.

**Figure 1.**
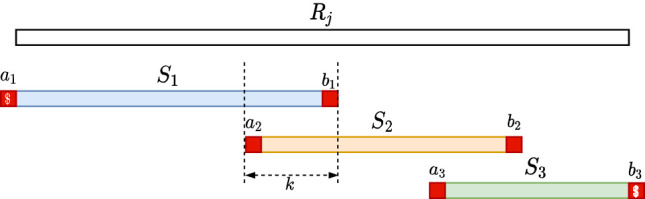
Illustration of the read splitting step. Read *R*_*j*_ is split into substrings *S*_1_, *S*_2_, *S*_3_ such that all *k*-mers of each *S*_*i*_ have the same minimizer, and extra linking characters (in red) are added to each *S*_*i*_. The overlap between two such consecutive extended *S*_*i*_s is of exactly *k* characters.

For every *S*_*i*_ obtained in this manner, let *a* and *b* be the characters of *R*_*j*_ immediately preceding and succeeding *S*_*i*_ in *R*_*j*_, respectively, or the character $ if they do not exist. We call *a* and *b linking characters*. Consider the string $S_{i}^{\prime }:=a\cdot S_{i}\cdot b$ and observe that $S_{i-1}^{\prime }$ and $S_{i}^{\prime }$ have a suffix–prefix overlap of *k* characters, because *S*_*i*−1_ and *S*_*i*_ have a suffix–prefix overlap of *k* − 2 characters and we added *b* at the end of *S*_*i*−1_ and *a* at the beginning of *S*_*i*_. For an illustration, see Figure 1. Recall that all (*k* − 1)-mers of *S*_*i*_ have the same minimizer, say *h*; we assign each extended string $S_{i}^{\prime }$ to a *group G*_*h*_ associated to such unique minimizer *h*. We say that a *k*-mer *x* appears in a group *G*_*h*_ if *x* is a substring of some $S_{i}^{\prime }$ in *G*_*h*_.

The above grouping strategy is similar to the one of Kokot et al. (2017), applied to *k*-mers instead of (*k* − 1)-mers (our strings $S_{1},\ldots ,S_{\ell_{j}}$ are called *super* (*k* − 1)*-mers* by Deorowicz et al. 2015), with the exception that when we group we add the linking characters. The following simple properties are key to ensuring the correctness of our approach.

Lemma 1. *Let x be a k-mer appearing in the reads R, and in a group G*_*h*_. *The following properties hold:*

- There is at most one other group in which *x* appears, and moreover, *x* appears in two distinct groups if and only if mini(pre_*k*−1_(*x*)) ≠ mini(suf_*k*−1_(*x*));
- occ(*x*, *G*_*h*_) = occ(*x*, *R*);
- If mini(*x*) = *h* (i.e., *x* does not contain a linking character) and it has a (*k* − 1) suffix–prefix overlap with some *k*-mer *y* (in either order), then also *y* appears in group *G*_*h*_.

Proof.

- Let *h*_1_: = mini(pre_*k*−1_(*x*)) and *h*_2_: = mini(suf_*k*−1_(*x*)). The *k*-mer *x* appears only in groups $G_{h_{1}}$ and $G_{h_{2}}$, which two distinct groups if *h*_1_ ≠ *h*_2_.
- This follows by construction of the group, because all the *k*-mer occurrences that have the same minimizer are put in the same group.
- If the minimizer of *y* is also *h* (i.e., it does not contain a linking character), then *y* also appears in *G*_*h*_. If not, recall that we added an extra character at the beginning and end of every string assigned to *G*_*h*_; thus, *y* is a *k*-mer containing a linking character and thus appears in *G*_*h*_.

Because we want to write the groups to disk and their number is the number of distinct minimizers, we merge the groups into a smaller number of buckets that are written to disk.

### Construction of intermediate (nonmaximal) unitigs

Lemma 1 ensures that extending any *k*-mer *x* can be correctly performed just by querying the group of *x*.

**Figure 2.**
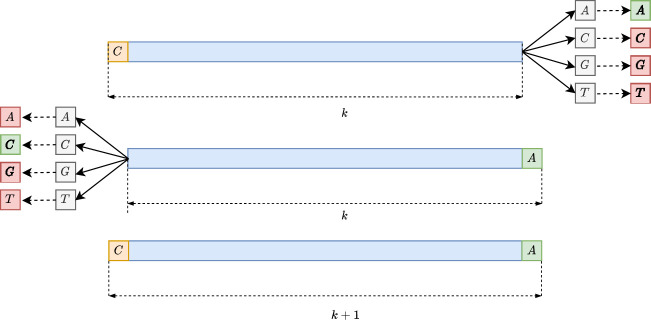
The extension step of the intermediate unitig construction happens inside each group. For each *k*-mer (*top*), it looks for a possible extension by checking all of the four possible neighbor *k*-mers in both directions and extends the *k*-mer (*bottom*) only if there is exactly one match both forward and backward (depicted in green in the first two figures from the *top*). Then it repeats the same process until no more extensions can be performed.

**Figure 3.**
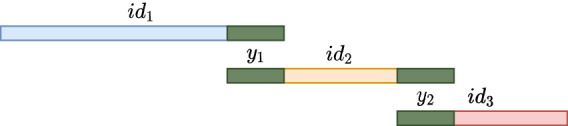
The result of the intermediate unitig construction. Each intermediate unitig that has a possible extension shares an ending with another intermediate unitig.

For each group, we perform the following:

1. A *k*-mer counting step of the strings in the group, using a hashmap, while also keeping track if a *k*-mer contains a linking character. More precisely, we scan each string in a group, and for each *k*-mer that we encounter, we increase by one its abundance in the hashmap and add a flag if it contains a linking character.
2. From the hashmap, we create a list of unique *k*-mers of the group that have the required abundance. This abundance check is correct thanks to Lemma 1(b).
3. We traverse the list of *k*-mers, and for each nonused *k*-mer *x*, we initialize a string *z*: = *x*, which will be extended right and left as long as it is a unitig (see Figs. 2, 3). We try to extend *z* to the right by querying the hashmap for suf_*k*−1_(*z*) · *c*, for all *c* ∈ {*A*, *C*, *G*, *T*}. If there is a unique extension *y* such that suf_*k*−1_(*z*) = pre_*k*−1_(*y*), then we query the hashmap for *c* · pre_*k*−1_(*y*), for all *c* ∈ {*A*, *C*, *G*, *T*}. If exactly one match is found (i.e., suf_*k*_(*z*)), then we replace *z* with $z{⊙}^{k-1}y$, and we mark *k*-mer *y* as used in the hashmap. If *y* is not marked in the hashmap as having a linking character, then we repeat this right extension with the new string *z*. The queries to the hashmap are correct thanks to Lemma 1(c). When we stop the right extension, we perform a symmetric left extension of *z*. After both extensions are completed, the resulting unitig *z* is given a unique index *id*_*z*_. If the extension of *z* was stopped because of a linking character in the first or last *k*-mer *y* of *z*, we add (*y*, *id*_*z*_) to a list *L*.

Notice that, after all groups have been processed, for any (*y*, *id*_*z*_) in *L*, there exist exactly one other $\left(y,id_{z^{\prime }}\right)$ in *L*, added from a different group, by Lemma 1(a). These two tuples indicate (nonmaximal) unitigs that have to be iteratively merged to obtain the maximal unitigs.

### Unitig merging

The tuples (*y*, *id*_*z*_) in *L* are sorted by *y*, such that the two entries (*y*, *id*_*z*_) and $\left(y,id_{z^{\prime }}\right)$ appear consecutively. Moreover, for any unitig *z*, there are at most two entries (*x*, *id*_*z*_) and (*y*, *id*_*z*_) in *L* (corresponding to its two end points). From these, we construct a list $\left(id_{z},id_{z^{\prime }}\right)$ that is put in another list *P* of pairs of unitig that must be merged into maximal unitigs. This is one of the hardest steps to parallelize, because no partitioning can be performed ahead of time that puts all the unitigs that are contained in a maximal unitig in the same partition. In other tools, for example, BCALM2 (Chikhi et al. 2016), this step is performed using a union-find data structure that can be difficult to be used with concurrency. Our solution uses a randomized approach (i.e., with guaranteed correctness and only *expected* running time) to put in the same partition the unitigs that should be merged, repeating the process until all the unitigs are merged into the final maximal unitigs.

**Figure 4.**
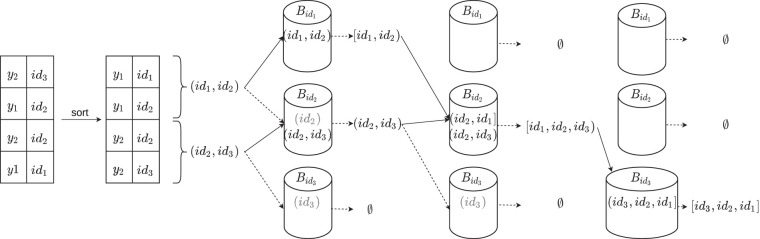
The unitig merging step on the unitigs from Figure 3. Each pair (ending, unitig index) is sorted by ending, and indexes of unitigs that share the same ending are joined in a tuple. Each such tuple is assigned to a bucket corresponding to one of its unsealed end point indices chosen at random (solid arrows in the figure). For the other end point index of the tuple (dashed arrow), we put a *placeholder* in its corresponding bucket (in gray). Then, inside each bucket, pairs sharing the same unitig index are joined to form larger tuples. If an ending cannot be joined and does not have a corresponding placeholder, then is it marked as sealed and is not selected anymore for bucket assignment. For example, in the first step, in $B_{id_{1}}$ the pair (*id*_1_, *id*_2_) is sealed at *id*_1_ because there is no placeholder for *id*_1_; however, in $B_{id_{2}}$ the pair (*id*_2_, *id*_3_) is not sealed at *id*_2_ because *id*_2_ has a placeholder. The steps are repeated until no more tuples can be joined. For the noncanonical case, we can merge the pairs if one extremity is at the end and the other one at the beginning of the pairs. For canonical *k*-mers, we also have to keep track of the direction of the *k*-mer before joining them; for more details, see the section “Construction correctness.”

We proceed as follows (see Fig. 4). We allocate a fixed number of buckets. Initially, for each list in *P*, we mark both its ends as *unsealed*. We repeat the following procedure until *P* is empty:

1. For each list in *P*, we choose at random one of its unsealed ends. Without loss of generality, let this end be *l*. We put the list in the bucket corresponding to *l*, whereas in the bucket corresponding to the other ending *r*, we put a placeholder.
2. Inside each bucket, we sort the lists by the ending that caused the list to be placed in the bucket. Then, we merge all the endings that are equal to produce longer lists. If an ending in a bucket is not merged and it has no corresponding placeholder (of another list) in the bucket, then it is marked as *sealed*.
3. Finally, we remove from *P* each list having both ends sealed.

In the above process, given two lists in *P* that must be merged, there is at least a probability of at least 1/4 that they are assigned to the same bucket to be merged (in the worst case, both ends are unsealed). Thus, in expectation, this desired outcome happens only after four tries.

Both *L* and *P* are also stored in buckets to allow a better concurrency while processing them.

### Construction correctness

We start by proving the correctness of the algorithm (without reverse complements).

Theorem 1. *Given a multiset R of strings, the strings U obtained at the end of our algorithm are the maximal unitigs of R*.

Proof. We prove that *U* satisfied the conditions of Definition 1 (for noncanonical unitigs).

For condition 0, because we start from *k*-mers, all strings in *U* have length at least *k*. To see that *U* is also a *set* (i.e., contains no duplicates), in our algorithm every *k*-mer is considered only once and is assigned to a unique unitig.

For condition 1, observe that the algorithm does not introduce any *k*-mer that is not also in *R*, and does not exclude any *k*-mer from *R*; thus, $\forall q\in \Sigma^{k}\;\mathrm{occ}(q,R)\geq 1\;\mathrm{iff}\;\mathrm{occ}(q,U)\geq 1$ holds. Lemma 1(b) guarantees that occ(*q*, *R*) the number of occurrences of *k*-mer *q* is the same as the number of occurrences in its group, and thus, the operation from the section “Construction of intermediate (nonmaximal) unitigs” performed inside its group respects its abundance in *R*. Thus, $\forall q\in \Sigma^{k}\;\mathrm{occ}(q,R)\geq a\;\mathrm{iff}\;\mathrm{occ}(q,U)\geq 1$.

Next, we prove conditions 3 and 2. Given an intermediary unitig *z*, we check for the eight *k*-mers that contain suf_*k*−1_(*z*); we extend *z* iff only two *k*-mers appear (one out-going from, and one in-coming to suf_*k*−1_(*z*)); thus, condition 3 is satisfied.

After merging *z* with this out-going *k*-mer, there are no other *k*-mers (and thus no other unitigs in *U* because each *k*-mer appears exactly once in *U*, because in the section “Construction of intermediate (nonmaximal) unitigs” we mark the used *k*-mers) that contain suf_*k*−1_(*z*) so condition 2 holds for single groups. This condition is still not satisfied globally, owing to the repetition of all the *k*-mers containing a linking character.

We now prove condition 2 after the unitig merging step. Note that the unitigs fed to the unitig merging step are the ones that start or end with a linking character, so they always overlap on *k* characters. After merging all the repeated *k*-mers, we satisfy condition 2 globally.

All the steps described so far can be easily adapted to work with canonical *k*-mers to obtain canonical maximal unitigs (Definition 1). This can be performed by changing two steps. First, the hash functions are replaced by their “canonical” version, such that the hash of a *k*-mer is always equal to the one of its reverse complement. Second, in the unitig merging steps, relative orientations of the unitigs are tracked to allow joining unitigs that can be present in opposite orientations in the input data set, by reverse-complementing one of them.

### Coloring

Computing the colors for each *k*-mer of a de Bruijn graph has two main challenges: (1) tracking all colors that belong to each *k*-mer and (2) storing the colors in a storage- and time-efficient manner.

To solve these two challenges, we propose a method partially inspired by the way BiFrost handles the colors, but with numerous improvements that allow for a smaller representation and a faster computation. The main idea is to merge color information for *k*-mers that share the same set of colors (see Almodaresi et al. 2017; Pandey et al. 2018; Mäklin et al. 2021), while avoiding costly comparisons of the entire sets for each *k*-mer. More precisely, for each *k*-mer, a normalized list *C* of colors is obtained by tracking the source of each *k*-mer, saving all the colors in a possibly redundant way (e.g., if the *k*-mer appears multiple times in a reference sequence), and then sorting and deduplicating them. From *C*, a 128-bit strong hash *h* is generated and is checked against a global hashmap that maps *h* to a color subset index. If a match is not found, then the list *L* is written to the color map, and a new incremental subset index for *L* is generated. Otherwise, it means that the color set already appeared in a previously processed *k*-mer, so the subset index of that color set is returned. Finally, the *k*-mer in the graph is labeled with its corresponding subset index, which, as discussed above, uniquely identifies a color subset. Overall, this allows a better compression, because each subset is encoded only once and not for every *k*-mer that belongs to it.

To optimize the disk space of the color map, this is encoded using a run-length compression scheme on the differences of the sorted colors of the subset, then it is divided into chunks for faster access and compressed again with a run of the lz4 algorithm. Furthermore, when writing the unitigs to disk, we mark the colors of each unitig in the header of the unitig sequence in the FASTA file by also run-length encoding the color set indices of all the *k*-mers of the unitigs. This strategy works well because most unitigs are “variation-free” and thus tend to have only a small number of possible color subsets associated to its *k*-mers.

### Sequence querying

GGCAT performs queries by dividing unitigs of the input graph and the queries in buckets, using an approach similar to the reads splitting step of the build algorithm. Then, independently for each bucket, a *k*-mer counting is performed to find the number of *k*-mers that match for each query. Finally, all the counters from different buckets are summed up to find for each query the number of *k*-mers that are present in the input graph. This also allows the partial matching of queries, because the output is the exact number of *k*-mer matches for each input sequence, and a percentage of required matching *k*-mers can be put as threshold to report a query as present. Similar to BiFrost, for the uncolored case, we return in output a CSV file with a line for each input query, containing the number and percentage of matched *k*-mers. For the colored case, we opted instead for a JSON Lines (JSONL) file with a line for each query, containing the number (if positive) of *k*-mer matches for each color *c* of the graph.

### Details of the data sets used in the evaluation

The Human Illumina read data set is the Illumina WGS 2 × 250-bp data set from the GIAB project, accession number HG004, https://github.com/genome-in-a-bottle/giab·data·indexes/blob/master/AshkenazimTrio/sequence.index.AJtrio·Illumina·2×250bps·06012016.HG004. The Human gut microbiome read data set was obtained from the NCBI BioProject database (https://www.ncbi.nlm.nih.gov/bioproject/) under accession number PRJEB33098 (Mas-Lloret et al. 2020). The 309 K *Salmonella* genome sequences were downloaded by us in February 2022 from the EnteroBase database (Zhou et al. 2020) and gzipped. The 100 Human genomes are from the set of 2505 Human genomes generated by Schmidt et al. (2023) using GRCh37 and the variant files from the 1000 Genomes Project (The 1000 Genomes Project Consortium 2015). For convenience we uploaded the Human genomes used for the benchmark to Zenodo (https://doi.org/10.5281/zenodo.2597496). The use of a newer Human genome reference would not significantly affect the conclusions of this study. Indeed, the computational performance of the tools are mainly dependent on the number of unique *k*-mers, which is mainly dependent on the variant files. However, in this setting, we would use the same variant files, even if a newer genome reference was used. The Bacterial genome sequences are from the data set used by Blackwell et al. (2021).

### Software availability

GGCAT is implemented in Rust and is available at GitHub (https://github.com/algbio/ggcat) and as Supplemental Code. For all tests, we used GGCAT commit f56da4d35f99f3537ec0a33f44d575898a8c91ea (https://github.com/algbio/ggcat/tree/f56da4d35f99f3537ec0a33f44d575898a8c91ea). The tools and scripts used to perform the benchmarks are available at GitHub (https://github.com/Guilucand/ggcat-test-benchmarks) and as Supplemental Code. The scripts for downloading the data sets used in the evaluation are available in the datasets-download/ folder of this repository.

## Supplementary Material

Supplement 1

Supplement 2
