# Supplementary material for: Extremely fast construction and querying of compacted and colored de Bruijn graphs with GGCAT
Source: Genome Res. 2023 Jul;33(7):1198–207. doi: 10.1101/gr.277615.122 (PMC10538363; doi:10.1101/gr.277615.122)
Supplement: Supplement 2 [file Supplemental_Methods.pdf]

Supplemental Material for
Extremely fast construction and querying of compacted and colored de Bruijn graphs with GGCAT

Andrea Cracco[1] and Alexandru I. Tomescu[2]

[1]*Department of Computer Science, University of Verona,* andrea.cracco@univr.it
[2]*Department of Computer Science, University of Helsinki,* alexandru.tomescu@helsinki.fi

Contents

1 Equivalence of node-centric and edge-centric unitigs **1**

2 Supplementary results **4**

3 Commands used **5**

3.1 Uncolored building 5
3.2 Colored building 5
3.3 Colored querying 5

1 Equivalence of node-centric and edge-centric unitigs

In this section we prove the equivalence between the (maximal) unitigs of the node-centric de Bruijn graph and the (maximal) unitigs of the edge-centric de Bruijn graph, built on the same set of strings, R, (i.e., their spellings are exactly the same strings). Note that we give this proof only for directed graphs.

For ease of notation, in this section we will denote the edge-centric graph of R as $G^e_k(R)$. The *node-centric* graph for R, which we denote as $G^n_k(R)$, is formally defined by adding a node for every k-mer of R, and an edge between two nodes x and y if $\mathsf{suf}_{k-1}(x) = \mathsf{pre}_{k-1}(y)$. In a node-centric graph, a path P (containing at least one node) is said to be a *node-centric unitig* if all nodes of P, except the last node, have out-degree equal to one, and all nodes of P, except the first one, have in-degree equal to one [1]. A node-centric unitig is said to be *maximal* if it cannot be extended by a node on either side [1]. See Supplemental Figure S1 for an example. We will use the term *unitig* to refer to a unitig in the edge-centric graph (as defined in the Preliminaries subsection of the main paper), and *node-centric unitig* to refer to the unitigs just defined in the node-centric graph.

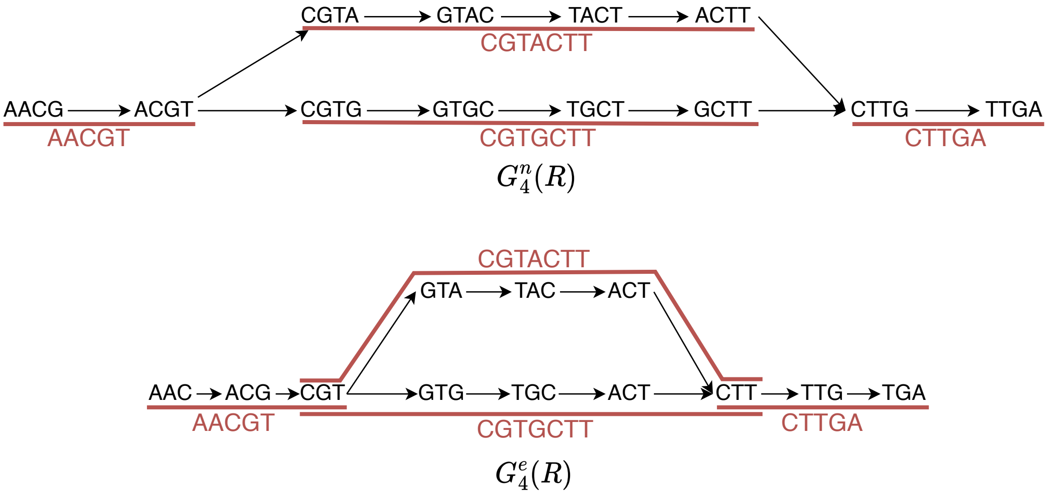

Supplemental Figure S1: Top: Illustration of the node-centric de Bruijn graph $G^n_4(R)$, where we assume R is the set consisting of all 4-mers that label the nodes of the shown graph. Bottom: the edge-centric de Bruijn graph $G^e_4(R)$, built on the same set R. In both graphs we draw in red their corresponding maximal unitigs (i.e., node-centric and edge-centric, respectively). In the node-centric graph, the node-centric unitigs in the middle of the figure do not include the node ACGT, since its out-degree is different from one, and do not include the node CTTG since its in-degree is different from one. Every unitig is labeled with its spelling, also in red. Notice that these spellings are the same in both graphs.

The *spelling* of a path $P = (x_1, \ldots, x_t)$ in $G^n_k(R)$ is analogously defined as the string $x_1 \odot^{k-1} \cdots \odot^{k-1} x_t$. As in the edge-centric case, by a node-centric unitig we will refer to either a path P in the node-centric graph, or to the spelling of P.

Theorem 1. *Let R be a set of strings, and let $\mathcal{X}$ be the set of all node-centric unitigs of $G^n_k(R)$ and let $\mathcal{Y}$ be the set of all unitigs of $G^e_k(R)$. Then $\mathcal{X} = \mathcal{Y}$.*

Proof. We prove the theorem by proving $\mathcal{X} \subseteq \mathcal{Y}$ and $\mathcal{Y} \subseteq \mathcal{X}$.

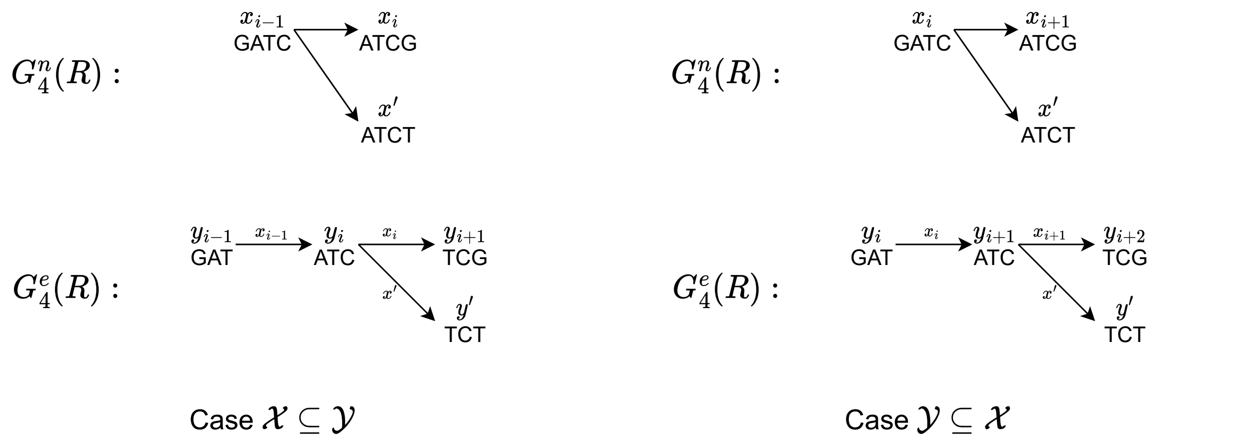

Supplemental Figure S2: Illustration of the two analogous cases in the proof of Theorem 1. For concreteness, we also draw some example strings on the nodes of graphs. In the edge-centric graphs on the bottom, we additionally label the edges with their corresponding k-mer.

$\mathcal{X} \subseteq \mathcal{Y}$: Let $X \in \mathcal{X}$; we show that $X \in \mathcal{Y}$ holds. Let $X = (x_1, \ldots, x_p)$, $p \geq 1$, and let $y_i := \mathsf{pre}_{k-1}(x_i)$, for each $i \in \{1, \ldots, p\}$, and let $y_{p+1} := \mathsf{suf}_{k-1}(x_p)$. Since $x_1, \ldots, x_p$ are k-mers of R, then the edges (y_i, y_{i+1}) exist in $G_k^e(R)$ for all $i \in \{1, \ldots, p\}$. We claim that $Y := (y_1, \ldots, y_{p+1})$ is a unitig in $G_k^e(R)$ (clearly, by construction, Y has the same spelling as X). First, note that Y contains at least one edge, since $p \geq 1$.

Suppose for a contradiction that Y is not a unitig in $G_k^e(R)$, namely that there is some internal node y_i, for some $i \in \{2, \ldots, p\}$, having in-degree or out-degree different than one; suppose w.l.o.g., that it has out-degree different than one. Since the edge (y_i, y_{i+1}) exists in $G_k^e(R)$, this means that the out-degree of y_i is non-zero, and thus at least two. Let (y_i, y') be another edge out-going from y_i ($y' \neq y_{i+1}$). Refer to Supplemental Figure S2(left) for an illustration of this configuration. Therefore, $x' := y_i \odot^{k-2} y'$ is a node in $G_k^n(R)$. Moreover, since $i \geq 2$, there is a node y_{i-1} preceding y_i in the unitig Y, and thus a node x_{i-1} preceding x_i in the unitig X. Consider now the nodes x_{i-1}, x_i, x' in $G_k^n(R)$. By definition, we have that $\mathsf{suf}_{k-1}(x_{i-1}) = y_i = \mathsf{pre}_{k-1}(x_i)$, and $\mathsf{suf}_{k-1}(x_{i-1}) = y_i = \mathsf{pre}_{k-1}(x')$. Thus, the out-degree of x_{i-1} in $G_k^n(R)$ is at least two. Since x_{i-1} is not the last node of the unitig X (since $i \leq p$), this contradicts the initial assumption that X was a node-centric unitig.

$\mathcal{Y} \subseteq \mathcal{X}$: Let $Y \in \mathcal{Y}$; we show that $Y \in \mathcal{X}$ holds. Let $Y = (y_1, \ldots, y_p)$, $p \geq 2$ (since edge-centric unitigs are defined to contain at least one edge), and let $x_i := y_i \odot^{k-2} y_{i+1}$, for each $i \in \{1, \ldots, p-1\}$. Since $x_1, \ldots, x_{p-1}$ are k-mers of R, then they are all nodes in $G_k^n(R)$, and $X := (x_1, \ldots, x_{p-1})$ (clearly, by construction X has the same spelling as Y) is a path in $G_k^n(R)$ (note that $p - 1 \geq 1$, since $p \geq 2$). We claim that X is a node-centric unitig in $G_k^n(R)$.

Suppose for a contradiction that this is not the case. W.l.o.g., we can assume that there is x_i, for some $i \in \{1, \ldots, p-2\}$, having out-degree different from one, and thus at least two. Let $x' \neq x_{i+1}$ be another out-neighbor of x_i, and let $y' := \mathsf{suf}_{k-1}(x')$. Refer to Supplemental Figure S2(right) for an illustration of this configuration. Consider now the nodes y_{i+1}, y_{i+2}, y' (recall $i \leq p-2$) in $G_k^e(R)$. By definition, (y_{i+1}, y_{i+2}) is an edge in $G_k^e(R)$; moreover (y_{i+1}, y') is also an edge in $G_k^e(R)$, because $x' = y_{i+1} \odot^{k-2} y'$ is a node in $G_k^n(R)$. Thus, the out-degree of the node y_{i+1} is at least two

in $G^e_k(R)$. Since $i \leq p-2$, this means that y_{i+1} is a node of the unitig Y, different from its last node y_p, having out-degree at least two, which contradicts the fact that Y is a unitig in $G^e_k(R)$. □

Next, we prove the same equivalence, but for *maximal* unitigs.

Corollary 1. *Let R be a set of strings, and let $\mathcal{X}$ be the set of all maximal node-centric unitigs of $G^n_k(R)$ and let $\mathcal{Y}$ be the set of all maximal unitigs of $G^e_k(R)$. Then $\mathcal{X} = \mathcal{Y}$.*

Proof. We prove one inclusion only, since the other one follows completely symmetrically. Let M be a maximal node-centric unitig in $G^n_k(R)$. We want to prove that M is also a maximal unitig in $G^e_k(R)$. Suppose for a contradiction that M is not maximal in $G^e_k(R)$. Then there exists a unitig M' of $G^e_k(R)$, $M \in M'$ with $|M'| > |M|$. By Theorem 1, we have that M' is also a node-centric unitig in $G^n_k(R)$. Since $M \in M'$, this contradicts the maximality of M in $G^n_k(R)$. Thus, M is a maximal unitig of $G^e_k(R)$. □

2 Supplementary results

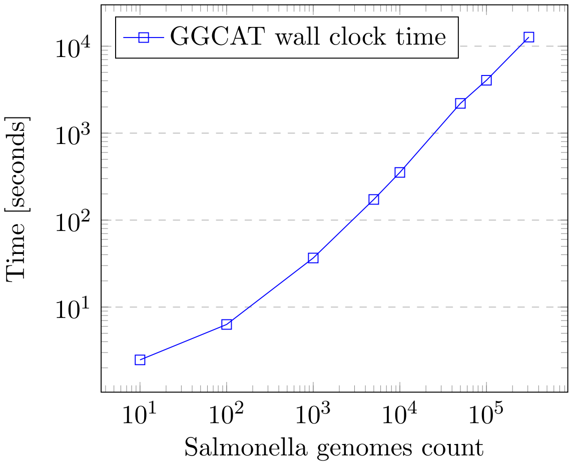

Supplemental Figure S3: GGCAT running time with an increasing amount of Salmonella genomes, $k = 63$, using 12 threads, on the small server.

Supplemental Table S1: Comparison of the colored index sizes. Here we exclude the size of the output fasta or GFA files containing the maximal unitigs, since they store the same data, and thus measure only the size of the index-supporting files of each tool.

Dataset	k	BiFrost colored	GGCAT colored
Human genomes (100)	27	2.00GB	0.09GB
	63	1.82GB	0.12GB
Salmonella genomes (100K)	27	52.62GB	33.10GB
	63	58.56GB	38.91GB

Supplemental Table S2: The default value of m (minimizer length) for each value of k (k-mer size) in a given range. These default values of m were chosen to give the best running time in the uncolored construction of the 1K Salmonella genomes.

k	m
< 13	$\max(k/2, k-4)$
14..15	9
16..21	10
22..30	11
31..37	12
38..42	13
43..64	14
> 64	$k/4$

3 Commands used

Here we list all the command templates used to benchmark the tools.

3.1 Uncolored building

```
# Cuttlefish 2 for sequencing reads
./cuttlefish build --read -l <INPUT_FILES_LIST> -k <KVALUE> -c <MULTIPLICITY> -t <THREADS> -o <OUTPUT_FILE> -w <TEMP_DIR>
# Cuttlefish 2 for reference genomes
./cuttlefish build --ref -l <INPUT_FILES_LIST> -k <KVALUE> -c <MULTIPLICITY> -t <THREADS> -o <OUTPUT_FILE>  -w <TEMP_DIR>

# GGCAT for both reads and reference genomes
./ggcat build -k <KVALUE> -j <THREADS> -s <MULTIPLICITY> -l <INPUT_FILES_LIST> -t <TEMP_DIR> -o <OUTPUT_FILE>
```

3.2 Colored building

```
# Bifrost
./Bifrost build -k <KVALUE> -t <THREADS>  <INPUT_FILES_LIST> -o <OUTPUT_FILE> --verbose -c

# GGCAT
./ggcat build -k <KVALUE> -j <THREADS> -s <MULTIPLICITY> -l <INPUT_FILES_LIST> -t <TEMP_DIR> -o <OUTPUT_FILE> --colors
```

3.3 Colored querying

```
# Bifrost
./Bifrost query -k <KVALUE> -t <THREADS> -g <INPUT_GRAPH> -q <INPUT_QUERY>  -o <OUTPUT_FILE> --verbose

# GGCAT
./ggcat query -k <KVALUE> -j <THREADS> <INPUT_GRAPH> <INPUT_QUERY> -t <TEMP_DIR> -o <OUTPUT_FILE> --colors
```

References

[1] Rayan Chikhi, Antoine Limasset, and Paul Medvedev. Compacting de Bruijn graphs from sequencing data quickly and in low memory. *Bioinformatics*, 32(12):i201–i208, 2016.
